# The Anti-Cancer Effects of Frondoside A

**DOI:** 10.3390/md16020064

**Published:** 2018-02-19

**Authors:** Thomas E. Adrian, Peter Collin

**Affiliations:** 1 Department of Physiology, Faculty of Medicine and Health Sciences, United Arab Emirates University, P.O. Box 17666 Al Ain, United Arab Emirates; 2 Coastside Bio Resources, Deer Isle, ME 04627, USA; pcollin48@gmail.com

**Keywords:** cancer, frondoside A, tumor growth, metastases, apoptosis, invasion, angiogenesis

## Abstract

Frondoside A is a triterpenoid glycoside from the Atlantic Sea Cucumber, *Cucumaria frondosa*. Frondoside A has a broad spectrum of anti-cancer effects, including induction of cellular apoptosis, inhibition of cancer cell growth, migration, invasion, formation of metastases, and angiogenesis. In cell lines and animal models studied to date, the anti-cancer effects of the compound are seen in all solid cancers, lymphomas, and leukemias studied to date. These effects appear to be due to potent inhibition of p21-activated kinase 1 (PAK1), which is up-regulated in many cancers. In mouse models, frondoside A has synergistic effects with conventional chemotherapeutic agents, such as gemcitabine, paclitaxel, and cisplatin. Frondoside A administration is well-tolerated. No side effects have been reported and the compound has no significant effects on body weight, blood cells, or on hepatic and renal function tests after long-term administration. Frondoside A may be valuable in the treatment of malignancies, either as a single agent or in combination with other therapeutic modalities.

## 1. Background

There is a desperate need for new and effective therapeutic agents for the treatment of cancer. It is particularly important to target growth, survival, migration, and metastases pathways in cancer with agents that have little or no toxicity towards normal cells. Over the years, there has been a search for such novel drugs in natural products. Many plant-derived compounds have been developed and used for treating cancer. Examples include doxorubicin, bleomycin, mitomycin, vincristine, and vinblastine. Marine organisms represent a vast untapped potential source of anti-cancer compounds and considerable effort has been invested in this area in recent years [1,2]. To date, there has been limited success in terms of marine-derived compounds or direct synthetic analogs of marine-derived compounds reaching the market for treatment of cancer and other disorders. The four notable exceptions for the treatment of cancer include cytarabine, trabectedin, eribulin mesylate, and monomethyl auristatin E (MMAE). Cytarabine, the pyrimidine nucleoside, 3-β-d-arabinofuranosylcytosine was synthesized following the discovery of arabinose-containing spongonucleosides from the sponge, *Cryptotethia crypta*. Cytarabine becomes incorporated into DNA in the place of a cytosine residue and halts DNA synthesis in the S phase of the cell cycle. It is used in the treatment of acute lymphocytic and myeloid leukemias, as well as chronic myeloid leukemia and non-Hodgkin’s lymphoma [1,2]. Gemcitabine, which is important in the treatment of pancreatic and non-small cell lung cancer, is a fluorinated analog of cytarabine. Trabectidine is a tetrahydroisoquinoline alkaloid produced synthetically. Trabectidine was originally isolated from the mangrove sea squirt *Ecteinascidia turbinata* but was subsequently shown to be produced by *Candidatus Endoecteinascidia frumentensis*, a microbial symbiont of the tunicate. Trabectidin inhibits activated transcription, notably of the drug resistance proteins, which are recognized to be the major pathways of resistance to chemotherapeutic drugs, such as doxorubicin and the taxanes [1,2]. Eribulin mesylate is a synthetic analogue of halichondrin B, a mitotic inhibitor from *Halichondria* sponges, which is used to treat patients with metastatic breast cancer or inoperable liposarcoma [2]. Monomethyl auristatin E (MMAE) is a synthetic highly potent antimitotic drug that is derived from peptides occurring in the marine shell-less mollusk, *Dolabella auricularia* called dolastatins. Because MMAE is highly toxic it is linked to a monoclonal antibody (MAB) that targets a membrane protein, CD30 found on anaplastic large cell lymphoma and Hodgkin’s lymphoma cells [2].

The search for anti-cancer compounds across different marine phyla has so far revealed several thousands of active compounds [2]. Of particular interest are echinoderms, which are phyla only found in the marine environment, which includes sea stars, sea urchins, sand dollars, sea cucumbers, and sea lilies. Sea cucumbers have been used in traditional Chinese medicine for treatment of cancer, inflammation, and other conditions for hundreds of years [3,4].

Triterpenoid glycosides from various sea cucumber species are known to have anti-cancer activity [5,6,7,8,9,10]. One particular triterpenoid glycoside, frondoside A has received particular attention, since it has shown potent anti-cancer effects in a broad spectrum of solid malignancies as well as in leukemias. Here, we will review the nature of frondoside A, its effects on cancer cell growth, cell cycle, apoptosis, angiogenesis, migration, invasion, and formation of metastases [11,12,13,14,15,16,17,18,19,20,21,22,23,24]. We will also review the pharmacokinetics, toxicity, interactions with other anti-cancer therapeutics, effects on the immune system, and possible mechanisms of action of this compound [13,16,21,22,23,24,25,26,27,28,29,30,31,32,33,34,35,36,37].

## 2. Structure of Frondoside A

**Figure 1:**
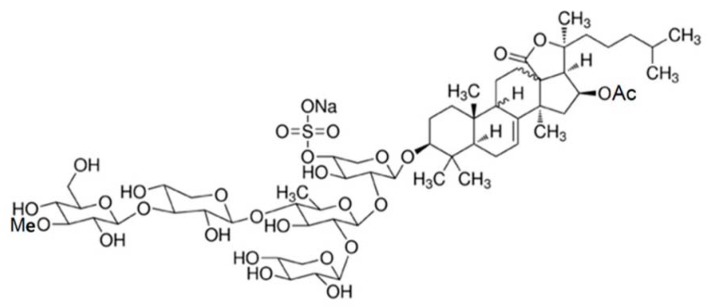
The structure of frondoside A.

Frondoside A is a triterpenoid glycoside with an acetoxy group at C-16 in the aglycone, which is a lanostane derivative. Frondoside A is a pentaoside with xylose as the third monosaccharide residue and 3-*O*-methylglucose as the terminal monosaccharide residue. It has a sulphate group on the first sugar residue. For the structure of frondoside A see Figure 1. Frondoside A differs from its closest cousin cucumarioside A_2_-2 (from *Cucumaria japonica*) in the functional group at C-16 of the aglycone (a keto group in cucumarioside A_2_-2) and the third carbohydrate group in the carbohydrate chain (glucose in cucumarioside A_2_-2). Frondosides B and C, also derived from *Cucumaria frondosa*, are found at slightly higher concentrations and have two and three sulphate groups, respectively, and some other minor structural differences compared with one in frondoside A (see Figure 1). Frondoside A has a molecular mass of 1334 Da.

The frondosides (A, B and C) can be readily isolated and purified, as previously described [28]. The resulting compounds have been shown to have high purity [11]. Frondoside A is extracted from either the freeze-dried cooking water from the sea cucumber processing plant or from freeze dried skin of the animal [28]. Briefly, the freeze dried powders are dissolved in chloroform/methanol [28]. Following evaporation, the extract is dissolved in water and mixed with ethyl acetate. After phase separation, the water phase is then loaded onto a Teflon column (DuPont 9B, Wilmington, DE, USA). The column is then washed with water to remove salts and pigments and the crude glycoside fraction eluted with 65% acetone. The glycosides are then purified on a Si 40 L 2632-2 flash column (Biotage, Charlotte, NC, USA) with the mobile phase mixture of chloroform/ethanol/water (100:100:17) as solvent. Purification is monitored by thin layer chromatography with 100:100:17 chloroform/ethanol/water as the solvent system. The frondoside A yield is approximately 0.1% of either starting material [28].

The ability of frondoside A to form supramolecular complexes with cholesterol was investigated using transmission electron microscopy [34]. The tubular nanoparticles that were detected were comprised of frondoside with cholesterol [34].

## 3. Effects of Frondoside A on Cancer Cell Proliferation and Viability

The effects of frondoside A on cell viability or proliferation has been tested using multiple different methods in many different cancers. This has included studies on pancreatic ductal adenocarcinoma, breast, non-small cell lung, colon, prostate, cervix, bladder (transitional cell), Burkitt lymphoma, malignant germ cell, and acute leukemias [11,12,13,14,16,18,19,20,21,23,24,25,26,27]. Methods employed have included cell counts, thymidine incorporation, MTT assay, and CellTiterGlo (Promega, Madison, WI, USA) assays. The results summarized as approximate IC_50_ are shown in Table 1. The IC_50_ for the effect of frondoside A from these studies across different cancer cell lines varies only between 0.1 and 3.0 µM. In the instances where the effect of frondoside A has been tested on immortalized non-malignant cell lines, these are much less responsive than the malignant cells, particularly when compared under identical conditions.

As mentioned above, several other sea cucumber-derived glycosylated triterpenoids have anti-cancer effects [5,6,7,8,9,10]. The activity of frondoside A was compared with frondosides B and C. In AsPC-1 and S2103 human pancreatic cancer cells studied in culture, frondoside A more potently reduced the number of viable cells than its disulphated cousin, frondoside B [26]. The trisulphated frondoside C and the parent aglycone had no effect on cell viability [26].

Frondoside A was submitted to the National Cancer Institute Developmental Therapeutics Program and was run twice through the NCI-60 cancer cell line screen. This screen includes leukemia and non-small cell lung, colon, CNS, melanoma, ovarian, renal, prostate, and breast cancer cell lines. Out of 57 cell lines investigated, frondoside A inhibited growth with ED_50_ below 1 µM for all but four cell lines (two melanoma, one renal, and one ovarian) (Coastside Bio Resources—unpublished data). 

## 4. Effects of Frondoside A on Cancer Growth

The results of studies of frondoside A undertaken in mice are summarized in Table 2. Frondoside A at an intraperitoneal (IP) dose of 10 µg/kg/day significantly reduced growth of AsPC-1 pancreatic cancer subcutaneous xenografts in athymic mice over a 32-day period [11]. No significant changes in body weights between frondoside A and vehicle control-treated animals were seen in these experiments [11]. 

In xenografts of MDA-MB-231 breast cancer cells the effect of frondoside A at 100 µg/kg/day IP was more effective than in the pancreatic cancer model [13]. Treatment began when the tumors averaged 200 mm^3^ in size. While tumors in the control group continued to grow exponentially, tumors in the frondoside A-treated group were reduced to almost nothing after treatment for 24 days [13]. Tumor weight was similarly dramatically decreased by frondoside A [13]. Again, there was no difference in body weight between treated and control animals. 

Subcutaneous frondoside A also inhibited the growth of LNM35 lung cancer cell xenografts [16]. By the end of a 10-day treatment period, frondoside A at a dose of 10 µg/kg/day IP had reduced tumor growth by more than 40% [16]. Increasing the IP frondoside A dose to 1000 µg/kg/day IP did not improve the efficacy of the compound, suggesting a narrow therapeutic window [16].

Frondoside A caused similar reductions in size of prostate cancer xenografts, using both PC-3 and DU145 prostate cancer cells [18]. For PC-3 cells treated with 100 µg/kg/day IP frondoside A, there was a modest inhibition of tumor growth over the 30-day treatment period [18]. Because they were less sensitive to the drug *in vitro*, animals with DU145 cells xenografts were treated with a higher dose (800 µg/kg/day IP) of frondoside A. This caused a more substantial reduction in tumor growth [18]. 

## 5. Effects of Frondoside A on Cell Cycle 

In prostate cancer cells, the effects of frondoside A appear to be cell line dependent. In cultured PC-3 cells, frondoside A caused a dose-responsive increase in cells arrested in the G2/M phase of the cell cycle and a reduction in the proportion in the G0/G1 phase [18]. In contrast, in DU145 and LNCaP cells no changes in the proportions of cells in the different phases were seen [18]. Similarly, there was no significant effect of frondoside A on cell cycle phase distribution in prostate cancer cells [19]. In four Burkitt lymphoma cell lines (BL-2, CA46, Namalwa, and Ramos) frondoside A caused a dose-responsive increase of cells in the G1 phase, with no significant changes in other phases [20].

## 6. Effects of Frondoside A on Programmed Cell Death

In cultured AsPC-1 pancreatic cancer cells, frondoside A was shown to induce apoptosis, as indicated by morphological changes, including cytoplasmic shrinkage, membrane blebbing, nuclear condensation, and loss of adhesion [11]. The induction of early and late apoptosis was confirmed by annexin V binding, which indicates the externalization of phosphatidylserine and by terminal deoxynucleotidyl triphosphate nick-end labeling (TUNEL) assay, indicating DNA fragmentation, respectively [11]. The apoptosis was associated with an increased expression of the pro-apoptosis protein, Bax, decrease in expression of the anti-apoptosis proteins, Bcl-2 and Mcl-1, and activation of caspase 3, 7, and 9 by cleavage [11]. In pancreatic cancer cells, frondoside A also resulted in a time-dependent increase in expression of the cyclin-dependent kinase inhibitor, p21. The increased expression of p21 is not a response to increased p53 activity, as p53 is mutated and inactive in the pancreatic cancer cells studied [11]. These findings indicate that in pancreatic cancer, frondoside A induces apoptosis via the mitochondrial pathway, while effects via death receptors were not investigated. 

In cultured MDA-MB-231 breast cancer cells, frondoside A induced apoptosis, as indicated by an increase in proportion of cells in the sub-G1 fraction in fluorescence-activated cell sorting analysis and by increased activity of caspase 3/7, as well as caspase 8 and caspase 9 [13]. Activation of caspase 3/7 was blocked by the caspase inhibitor, Z-DEVD-FMK [13]. In studies by the same group, frondoside A induced a similar increase in caspase 3/7 activity in LNM35 lung cancer cells [16]. 

In cultured PC-3, DU145 and LNCaP prostate cancer cells, frondoside A was shown to induce apoptosis by both the sub-G1 fraction during cell cycle analysis and increase in annexin V binding [18]. The pan-caspase inhibitor, Z-VAD-FMK significantly decreased induction of apoptosis in DU145 cells, but not in PC-3 of LNCaP cells [18]. The apoptosis induced by frondoside A was accompanied by induction of caspase 3 and poly (ADP-ribose) polymerase (PARP) cleavage and activation, upregulation of the pro-apoptosis factors Bax and PTEN or Bad and downregulation of the anti-apoptosis protein Bcl-2 [18]. In PC-3 and DU145 cells, frondoside A increased the level of phosphor-mTOR and expression of p21. In contrast, expression of p21 was decreased in frondoside A-treated LNCaP cells [18]. In addition, frondoside A also inhibited pro-survival autophagy in prostate cancer cells [18].

In the bladder urothelial carcinoma cell line, RT112 frondoside A induced caspase-independent apoptosis. Frondoside A induced a concentration-dependent increase in expression of Bax and p21, activation of caspases 3, 8, and 9, PARP cleavage, and DNA fragmentation [19]. The induction of apoptosis was not affected by pre-treatment with the pan-caspase inhibitor, Z-VAD-FMK [19]. As in pancreatic cancer, the increase in p21 expression was not driven by a change in p53 and inhibition of p53 activity did not suppress frondoside A induced cell death [19]. As in prostate cancer cells, frondoside A inhibited pro-survival autophagy in RT112 cells with time and concentration-dependent accumulation of the autophagy-related proteins, LC3B-II and p62 and accumulation of cellular autophagosomes [19].

In Burkitt lymphoma cell lines, CA46, Namalwa, Ramos, and BL-2, frondoside A induced phosphatidyl serine externalization, caspase-3 activation, decreased expression of BCl-2 and survivin, increased the cytoplasmic content of cytochrome C and apoptosis-inducing factor (AIF), as well as DNA fragmentation, indicating apoptosis [20]. However, again the pan-caspase inhibitor, Z-VAD-FMK did not diminish frondoside A-induced apoptosis in any of the tested cell lines, indicating that the induction of apoptosis was not caspase-dependent [20]. Similarly, frondoside A inhibited pro-survival autophagy in RT112 cells with time-dependent accumulation of the autophagy-related proteins, LC3B-II and SQSTM1/p62 [20]. Furthermore, the effects of frondoside A were independent of p53 status and the apoptosis induction was not antagonized by p53 inhibition [20]. 

*In vitro* treatment of NCCIT and 2102EP germ cell tumor lines resulted in caspase-independent apoptosis and the use of the caspase inhibitor, Z-VAD-FMK confirmed that extensive apoptosis occurred despite caspase inhibition [24]. As in the Burkitt lymphoma cells, the apoptosis was associated with increased accumulation of AIF, and again frondoside A inhibited pro-survival autophagy [24]. 

In HL60, THP-1, and NB4 human leukemia cells, frondoside A induced time and concentration-dependent apoptosis, as indicated by annexin V binding [12]. In HL60 cells, apoptosis was not associated with a change in mitochondrial permeability or cytochrome C release into the cytoplasm. However, after six hours of frondoside A treatment there was an increase in the activation of caspases 3, 7, 8, and 9, and cleavage of PARP [12]. Depending on the time point or concentration of frondoside A, pretreatment of HL60 cells with caspase inhibitors, Z-DEVD-FMK or Z-VAD-FMK had little or no effect on the induction of apoptosis, indicating once again that the apoptosis in leukemia cells was caspase-independent [12]. 

For many years, caspase-dependent apoptosis was considered synonymous with programmed cell death, however it has become evident in recent years that there are caspase-independent forms of programmed cell death. It is likely that alternate or backup pathways evolved as the caspase-dependent pathway could be circumvented by viruses or cell transformation. A classification of different pathways of programmed cell death was proposed by Leist and Jäättelä [37]. Their classification was based on both morphological and biochemical criteria and included three forms of programmed cell death in addition to classical necrosis. The first was the classical, caspase-dependent apoptosis with cell shrinkage, membrane blebbing, chromatin condensation, phosphatidylserine relocation to the outer cell membrane, activation of the caspase cascade, and internucleosomal DNA cleavage [37]. The second was apoptosis-like cell death with less compact chromatin condensation, phosphatidylserine translocation, but without activation of the caspase cascade [37]. The third was necrosis-like cell death occurring in the absence of either chromatin condensation or caspase activation [37]. In addition, it is now clear that there are other specialized forms of programmed cell death not fitting into the above models, including paraptosis and dark cell death [38,39]. Finally, another form of programmed cell death is autophagy, characterized by marked cytoplasmic vacuolization, where cellular components are destroyed through an autophagosomic-lysosomal pathway [40]. 

Activation of the death receptor pathway by the binding of tumor necrosis factor-α associated ligands or the Fas ligand to their respective receptors can induce either classical apoptosis or necrosis-like cell death, depending on the experimental conditions [41,42]. In addition, knockout studies have revealed that necrosis-like cell death triggered through the death receptor pathway requires Fas-associated death domain (FADD)-mediated activation of the protein kinase receptor interacting protein (RIP), which activates nuclear factor kB (NF-κB) [41]. The molecular mechanisms of death receptor-mediated cell death have not been completely characterized, but mitochondrial dysfunction and non-caspase-proteases appear to be involved in this process [43,44,45]. In the presence of caspase inhibitors, death receptor-mediated necrosis requires a mitochondrial step, but Bid cleavage and mitochondrial cytochrome c release are not involved [43,44]. In contrast, necrosis-like cell death is associated with the increased production of mitochondrial reactive oxygen species and antioxidants can block this form of cell death [41,42,43,46]. Thus, there are several possible mechanisms to account for the observed caspase-independent programmed cell death that is seen in several of the frondoside A-treated cell lines.

Extensive studies have been carried out using caspase inhibitors, particularly the broad-spectrum caspase inhibitor, Z-VAD-FMK. These studies have revealed that apoptosis can be slowed but never completely prevented by the inhibitor, suggesting that caspase-dependent and independent apoptosis pathways may be triggered simultaneously [47]. Indeed, it has been proposed that no experimental system exists where Z-VAD-FMK can prevent cell death [48]. This has been tested with many different apoptosis triggers and induction of apoptosis through both the death receptor-mediated and intrinsic mitochondrial pathways [47,48,49,50,51]. It is also possible that pan-caspase inhibitors, such as Z-VAD-FMK do not completely inhibit activity of all pro-apoptotic caspases. Furthermore, their inhibition of other proteases, such as calpains and cathepsins may add to the difficulty in interpreting experimental data. Caspase-independent apoptosis pathways are likely to involve other mitochondrial proteins, such as apoptosis-inducing factor (AIF), as well as other cellular proteases [52]. AIF is found in the slime mold, *Dicostelium discoideum,* which predates the evolutionary development of caspases [53]. Non-caspase proteases that appear to be involved in programmed cell death include granzymes A and B, HtrA2, which is released from mitochondria, cathepsins B and D, and calpains [48].

## 7. Anti-Angiogenic Effects of Frondoside A

Frondoside A has been shown to have antiangiogenic effects in the chick chorioallentoic membrane (CAM) assay, inhibition of vascular tube formation in cultured human umbilical vein endothelial cells (HUVEC), and in xenografts of human lung tumors [16]. In the CAM assay, frondoside A caused concentration-dependent inhibition of basal angiogenesis at concentrations as low as 100 and 500 nM [16]. Furthermore, frondoside A (500 nM) completely abolished the increased angiogenesis that was triggered by basic fibroblast growth factor (bFGF, 2 µg/L [16]. When cultured on Matrigel-coated plates, HUVEC cells spontaneously form vascular tube-like structures. Frondoside A (500 nM) almost completely abolished vascular tube formation at a concentration of (500 nM), but had no significant effect on the viability of the HUVEC cells at this concentration, indicating a lack of cytotoxicity on the HUVEC cells [16]. Microvessel density, measured by CD31 immunohistochemical staining in the periphery of xenografted tumors, was markedly reduced by frondoside A in animals treated with a dose of 10 µg/kg/day IP [16].

## 8. Effects of Frondoside A on Migration and Invasion

Progression of cancer is associated with loss of the normal constraints on cellular migration and invasion. Frondoside A has been shown to inhibit migration and invasion of both breast and lung cancer cells [13,16]. Migration is measured microscopically in the wound-healing model, where a 1 mm scrape is made with pipette tip though a confluent monolayer of cells, as cells move in to fill the gap. Frondoside A caused concentration and time-dependent inhibition of migration of MBA-MD-231 breast cancer cells and LNM35 lung cancer cells at concentrations (0.1–1.0 µM) that have no effect on viability during the time interval of the wounding assay [13,16]. The effect of frondoside A on invasion was measured using the Matrigel invasion assay in the same cell lines. Frondoside A caused concentration-dependent inhibition of invasion over a 24-hour period [13,16]. Marked inhibition of invasion was seen at frondoside A concentrations (0.1–0.5 µM) that had little or no effect on cell viability [13].

In a separate study in MDA-MB-231 cells, frondoside A inhibited TPA-induced colony formation, migration, and invasion associated with reduction in the expression, secretion, and enzymic activity of matrix metalloproteinase-9 (MMP-9), enhanced expression of tissue inhibitors of metalloproteinases 1 and 2 (TIMP-1 and TIMP-2), as well as reduced activation of activator protein-1 (AP-1, a heterodimer of c-Fos and c-Jun) and nuclear factor kappa B (NF-κB) transcription factors [15]. These findings suggest that the inhibition of invasion is mediated via the changes in these factors [15].

Frondoside A has been shown to inhibit formation of metastases in breast, lung, and prostate cancers [14,16,18]. For example, breast cancer metastases were investigated after 66.1 mouse mammary cancer cells, pretreated with frondoside A, or control vehicle, for 30 minutes, were injected into the tail vein of mice and the spontaneous development of lung metastases counted after three weeks. In this model, pretreatment with 5 µM frondoside A IP reduced the number of lung tumor colonies by 45% [14]. In a separate experiment, 1 µM frondoside A IP also markedly reduced the formation of metastases, while exposure to 0.1 µM frondoside A was ineffective [14]. In a more clinically relevant model, the 66.1 cells were implanted subcutaneously proximal to the right mammary gland of mice and frondoside A treatment was administered IP each day for 10 days. Formation of spontaneous metastases was significantly inhibited by frondoside A at 50 µg/kg/day and even at 10 µg/kg/day the inhibition almost reached statistical significance (*p* < 0.06) [14].

A separate study investigated the effect of frondoside A on development of axillary lymph node metastases after subcutaneous implantation of LNM35 lung cancer cells [16]. Frondoside A at a dose of 10 µg/kg/day IP significantly reduced the average weight of the lymph nodes by more than 50%; however, a higher dose of 1 mg/kg/day was no more effective [16].

In xenografts comprised of PC-3 prostate cancer cells, frondoside A markedly reduced the number of lung metastases and caused a similar decrease in the presence of cancer cells detected using a human DNA detection method [18]. With DU145 cell xenografts, there were no lung metastases detected by microscopy and there was a marked reduction in the tumor cell detection in lung using the human DNA detection method; furthermore, frondoside A significantly reduced the detection of tumor cells in the blood using this method [18].

## 9. Effects of Frondoside A on Multidrug Resistance

Development of resistance of cancer cells to antitumor drugs with completely different mechanisms of action is a well-known phenomenon, known as multidrug resistance (MDR) [35]. The major mechanism of multidrug resistance is through the upregulation of transmembrane transport proteins that efflux drugs from the cells, lowering the intracellular concentrations of drugs and rendering them ineffective. The major drug efflux protein is known as permeability glycoprotein (P-glycoprotein). Activity of P-glycoprotein can be measured by efflux of fluorescein dyes, which enter the cells by diffusion through the cell membrane. Frondoside A, or nanoparticle complexes of frondoside A with cholesterol can block P-glycoprotein activity [35]. Inhibition of MDR was seen with a frondoside A concentration of only 750 pM (1 ng/mL) and no greater effect was seen when concentrations were increased to 7.5 or 75 nM. On a molar basis, frondoside A was more effective than verapamil, the most effective concentration of which was 26.4 nM (12 ng/mL) [35].

## 10. Interactions with Other Anticancer Drugs

The results of the following studies are summarized in Table 2. In MDA-MB-231 cells in culture, frondoside A enhanced the anti-proliferative effects of paclitaxel, a drug that targets tubulin and prevents microtubule formation, in an apparently additive manner [13]. The combination of frondoside A with cisplatin, a drug which inhibits DNA replication, was tested in the mouse xenograft model with LMN35 lung cancer cells [16]. When administered daily, each of these drugs alone inhibited tumor growth and by the tenth day tumor size was about 40% lower in the treated groups. The combination of the two, however, suppressed tumor growth by 68% (*p* < 0.05) [16]. Combinations of frondoside A with both cisplatin and gemcitabine (another drug that impairs DNA synthesis) were tested in RT112 urothelial cancer cells [19]. Both drug combinations had marked synergistic effects in these cells [19]. Because frondoside A has similar growth inhibitory effects in cancer cells regardless of their p53 status, a study was conducted to investigate the effects of frondoside A and cisplatin after pretreating cells with pifithrin-α (Pif-α), which is a chemical inhibitor of p53 transcriptional activity in wild-type p53 BL-2 Burkitt lymphoma cells [20]. There was a clear additive effect of frondoside A and Pif-α in these experiments, while the effect of cisplatin was antagonized by Pif-α [20]. These findings indicate that while cisplatin activity is p53-dependent, functional p53 is not required for the anti-cancer activity of frondoside A [20].

Studies in AsPC-1 and S2013 pancreatic cancer cells revealed marked synergistic effects of low concentrations of frondoside A with gemcitabine in cell culture [25]. Furthermore, the combination also showed enhanced effects compared with either drug alone in the xenograft model of pancreatic cancer using either cell line [25]. 

Frondoside A also potentiates the actions of several conventional therapeutic agents in acute leukemia cell lines [21]. Frondoside A enhanced the anti-leukemic effects of vincristine, asparaginase, and prednisolone in CCRF-CEM, THP-1, and HL-60 cells [21]. Synergistic effects were seen with frondoside A in combination with each of the three other drugs in CCRF-CEM and THP-1 cells [21].

## 11. Effects of Frondoside A on the Immune System

Frondoside A exhibits a range of very potent immunomodulatory effects *in vitro* and in animals. While the observed effects of frondoside A in the human xenograft models in athymic mice are clearly not related to effects on the immune system, such immunological effects may contribute to the anti-cancer effects of the compound in other animal tumor models and could potentially contribute an additive effect if frondoside A becomes used as a drug in humans. 

Frondoside A potently stimulates lysosomal activity in mouse macrophages *in vivo* [28]. The maximal stimulatory effect was seen with 0.2 µg/mouse and the effect was maintained for 10 days [28]. This dose is similar to the lowest dose (10 µg/kg/day) that has shown anti-cancer activity in athymic mice [16], but it is intriguing that the effect lasts for ten days after a single dose [28]. Frondoside A also stimulates an increase in the number of antibody plaque-forming B-cells in the spleen of mice in immunized with sheep erythrocytes, again with a maximal effect seen at a dose of 0.2 µg/mouse [28]. Frondoside A also had a weak effect on IgM production in response to the immunization with sheep erythrocytes. However, frondoside A had no effect on immunoglobulin production in mice immunized with ovalbumin [28]. Frondoside A stimulated lysosomal activity in mouse macrophages by 30% *in vitro* at concentrations of 75–285 nM (0.1–0.38 µg/mL) [28]. Frondoside A very potently enhances macrophage phagocytosis of the bacterium Staphylococcus aureus and stimulates production of reactive oxygen species *in vitro* at a maximal effective concentration of 750 pM (1 ng/mL) [28]. Hence, frondoside A is an immunostimulant of cell-based immunity including phagocytosis without significant amplification of humoral immune activity or adjuvant properties and may be valuable in treating disorders where depleted immune status contributes to the pathological process [28].

One study investigated the protein changes that occurred in frondoside A stimulated splenocyte cultures using proteomics [29]. Thirty proteins were differentially expressed, including down-regulation of Septin-2, a protein that hetero-oligomerizes with other septins to form filaments. Loss of Septin-2 causes actin stress fibers to disintegrate and cells to lose polarity. Other down-regulated proteins include NADH dehydrogenase iron-sulfur protein 3 (an enzyme which is a component of mitochondrial NADH: ubiquinone oxidoreductase), and GRB2-related adaptor protein 2 (an adaptor-like protein involved in leukocyte-specific protein-tyrosine kinase signaling) [29]. Up-regulated proteins include N-ethylmaleimide-sensitive factor-like 1 cofactor p47 (a protein necessary for the fragmentation of Golgi stacks during mitosis and for their reassembly after mitosis), and heterogeneous nuclear ribonucleoprotein K (a nucleic acid-binding protein that serves as a docking platform integrating transduction pathways to nucleic acid -directed processes) [29]. Together with the results of proliferation and adhesion assays, these changes suggest that in addition to stimulating splenocyte proliferation, frondoside A has immunostimulatory effects that enhance the cellular defense mechanism necessary to fight pathogens for which lymphocytes and splenocytes need to be recruited [29].

Another study revealed that frondoside A inhibits the non-specific esterase of mouse spleen lymphocytes, but the concentrations for this inhibitory effect was higher than required for the immunomodulatory effects [30].

In macrophages, frondoside A stimulates spreading, lysosomal activity, and the formation of reactive oxygen species [31].

Prostaglandin E_2_ (PGE_2_) from tumor cells inhibits natural killer (NK) cell functions. Indeed, several functions of these cells, including lysis, migration, and cytokine production, are compromised in tumor bearing mice. Similarly, PGE_2_ prevents migration, the cytotoxic effects, and interferon γ (IFNγ) production in cultured NK cells. Frondoside A, which acts as a blocker for EP_4_ prostaglandin receptors, inhibits breast cancer metastases in an NK cell-dependent manner and protects IFNγ production from NK cells from PGE_2_ mediated suppression [32].

## 12. Pharmacokinetics of Frondoside A and Route of Administration

The pharmacokinetics of frondoside A were investigated in mice following intravenous (IV) and intraperitoneal (IP) administration at a bolus dose of 100 µg/kg. Plasma frondoside A concentrations were measured using a liquid chromatography mass spectrometry (LC-MS/MS) method [26]. The mean C_max_ following IV administration of frondoside A was 129nM, while that following IP administration was 18.3 nM at 45 min, which is about seven-fold lower than with IV administration at the same dose [26]. The calculated bioavailability after IP administration was approximately 20%. Following IV administration, plasma concentrations of frondoside A remained above 7.5 nM for 17 h, while for IP administration, plasma levels remained above this level for only 4 h [26]. In contrast, oral dosing resulted in very low and variable plasma concentrations of frondoside A near to or below the detection limit of the assay. The half-life of IV administered frondoside A was 8.5 h [26].

The low plasma concentrations of frondoside A after oral administration were confirmed when the effect on growth of AsPC-1 xenografts was compared with IP administration in athymic mice. While IP administration resulted in near to complete inhibition of tumor growth, oral administration was completely ineffective with the time course being almost identical to the vehicle control. These findings suggest very low bioavailability from the oral administration of frondoside A, which is likely to reflect either poor absorption or rapid digestion in the intestine. Indeed, since the aglycone showed no anti-cancer effect, it is likely that the glycosyl groups are cleaved by digestive enzymes, resulting in an inactive aglycone [26]. 

## 13. Toxicity of Frondoside A

The reported studies of frondoside A *in vivo* have failed to show any hint of a toxic effect at the studied doses, which are up to 1000 µg/kg/day [11,13,14,16,18,25]. There are no apparent side effects and body weight, liver function, and hematological parameters are not adversely affected by the drug. In a study of athymic mice with MDA-MB-231 cell xenografts, frondoside A administered at 100 µg/kg/day had absolutely no effect on numbers of white blood cells, red blood cells, platelets, or hemoglobin, or on plasma concentrations of blood urea nitrogen, creatinine, aspartate aminotransferase, or alanine aminotransferase [13]. A study in mice with xenografts of PC-3 prostate cancer cells revealed no significant changes in hemoglobin, WBC, lymphocyte, monocyte, neutrophil, or platelet counts with frondoside A at a dose of 100 µg/kg/day [18]. At a higher dose of 800 µg/kg/day, frondoside A caused non-significant increases in WBC, lymphocyte, and neutrophil counts, but a significant (*p* < 0.01) increase in monocyte count in mice with DU145 prostate cancer xenografts [18]. A formal study of the toxicity of frondoside A revealed that the LD_50_ in mice was 9.9 mg/kg, which is 100-fold greater than the dose used in most of the *in vivo* experiments testing efficacy [28].

## 14. Mechanisms of Action

Up until now, the mechanism by which frondoside A triggers its anti-cancer and other effects has been somewhat of a mystery and several possible mechanisms have been proposed, however new evidence reveals a unifying hypothesis that can account for most, if not all, of the observed biological mechanisms.

Acting as potent inhibitor of the multi-drug resistance, G-glycoprotein would certainly be valuable in the treatment of cancer, regardless of the mechanisms that mediate the effects of frondoside A on proliferation, cell cycle, apoptosis, migration, invasion and angiogenesis [35]. 

Because of the steroid backbone of the molecule, an early study investigated whether frondoside A had estrogenic activity using a yeast two-hybrid system [33]. No appreciable estrogenic activity was detected [33].

In a study that showed marked effects of frondoside A on the inhibition of 66.1 mouse mammary cancer cell growth and the development of metastases revealed that the compound also blocked binding and activation of the EP_4_, and to a lesser extent, EP_2_ prostaglandin receptors [14]. Frondoside A caused inhibition of tritiated PGE_2_ from binding to EP_4_ receptors with an IC_50_ of approximately 3.7 µM and EP2 receptors with an IC_50_ of approximately 16.5 µM [14]. Frondoside A was also able to cause concentration-dependent inhibition of PGE_2_-stimulated intracellular cyclic AMP concentrations [14]. Complete inhibition of PGE_2_-stimulated intracellular cyclic AMP was seen at a frondoside A concentration of 5 µM [14]. Curiously, in the absence of PGE_2_, frondoside A at a concentration of 1 µM caused an increase in intracellular cAMP levels, almost rivalling that of PGE_2_ [14]. Furthermore, it is notable that the IC_50_ for the effect of frondoside A on cell proliferation was 0.5 µM, almost eight-fold lower than that for inhibition of PGE_2_ binding to EP_4_ receptors and 33-fold lower for EP_2_ receptor binding [14]. These findings suggest that even if prostaglandin receptor blockade contributes to the anti-cancer effects of frondoside A, other more potent mechanisms are likely to be involved.

Another study, which was designed to investigate the mechanism of action, employed microarray using a human oligonucleotide expression array library coupled with real-time RT-PCR to study the transcriptome of S2013 pancreatic cancer cells treated with 2μM frondoside A for 6h as compared with that of untreated cells [52]. Expression of genes showing the greatest changes were confirmed by real-time RT-PCR and time-courses of gene expression were investigated in seven cancer cell lines. Marked changes were seen in expression of several genes involved in growth regulation. Downregulated genes included E2F1, cyclin A2, cdc20, cdc21, cdc45, and cdc47, all of which play important roles in DNA replication and cell cycle control [54]. Upregulated genes included fatty acid binding protein 3 (FABP3), growth and development factor 15 (GDF15), p21^WAF-1^ (which has been shown to be upregulated in multiple studies as outlined above), repressor of E1A, dual-specificity phosphatase, and death-associated protein kinase-1 [54]. Attention was focused on GDF15 and FABP3 [54]. GDF15 belongs to the transforming growth factor superfamily that plays a role in regulating inflammatory and apoptotic pathways during tissue injury, and mediates apoptosis induction in response to NSAIDS. FABP3 is a candidate tumor-suppressor that arrests growth of mammary epithelial cells. Knockdown of expression of either GDF15 or FABP3 using specific siRNA in AsPC-1 cells reversed the growth inhibitory effects of frondoside A. These findings suggest that both GDF15 and FABP3 are involved in the growth inhibitory effects of frondoside A in pancreatic cancer. Since this mechanism appears unique, it explains the synergistic anti-cancer effects seen when combined with other agents, such as cisplatin, paclitaxel, and gemcitabine.

In a study investigating the effects of frondoside A in migration and invasion of breast cancer cells, it was revealed that frondoside A could inhibit TPA-induced activation of MMP-9 via pathways involving inhibition of activation of two transcription factors, AP-1 and NF-κB [15]. Furthermore, frondoside A reduced the ATP-stimulated phosphorylation of several kinase pathways, including phosphoinositide 3 kinase/protein kinase B pathway (PI3K/Akt), the extracellular signal-regulated kinases (ERK1/2), and p38 mitogen activated protein kinase (p38 MAPK), which are all involved in growth stimulatory and cell survival pathways [15]. This study provided valuable insight into how frondoside A could be having widespread effects in the inhibition of cell growth, cell survival, migration, invasion, metastasis, and angiogenesis.

A breakthrough in our understanding of the mechanisms by which frondoside A has such widespread effects came recently when a paper revealed that it was a potent inhibitor (IC50 1.2 µM) of RAC/CDC42-activated kinase (PAK1), with an IC_50_ around 1.2 µM *in vitro* (not in cell culture) [36]. Furthermore, its direct action is highly specific for PAK1, because IC_50_ for other kinases such as LIM kinase and AKT is around 60 μM [36]. This potency is in line with the anticancer effects of frondoside A, which from multiple studies is approximately 1 µM (see Table 1). The discovery that frondoside A inhibits PAK1 is unifying since this kinase is upstream of several other transduction mechanisms, including Ap-1 and NF-κB, already implicated in the actions of the compound [15]. Furthermore, PAK1 is involved in stimulating cancer cell growth, invasion, and metastasis [55,56]. PAK1 activation also potently increases angiogenesis and tumor cell-survival autophagy [36,57]. So being a PAK1 inhibitor may explain the broad spectrum of biological actions on tumors, including inhibition of growth, migration, invasion, metastasis, angiogenesis, and pro-survival autophagy, as well as the induction of apoptosis. Expression of the p21 gene is suppressed by PAK-1 and is increased by frondoside A [36,58].

The major aspect of the biological effects of frondoside A that cannot be readily explained by PAK-1 inhibition are the immunomodulatory effects of the compound [28,29,30]. PAK1 appears to act as an immuno-suppressor, and either PAK1 si-RNA or chemical PAK1-inhibition boosts the immune response in mice [59]. However, the *in vitro* effects are seen at concentrations that are much lower than those that have either anti-cancer or PAK-1 inhibitory effects and the *in vivo* effects are seen with very low doses of the compound [28,29]. One possible explanation is that the immunomodulatory effects are mediated by a metabolite of frondoside A, perhaps its aglycone, which is likely to be absorbed intact from the gut. Alternatively, perhaps the immune modulatory effects are mediated by action in the gut and do not require absorption of the compound. A recent study demonstrated that curcumin, another compound with poor oral availability but potent effects on the attenuating arthritis, was mediated by increasing neuroexcitability of the vagus nerve [60]. It is interesting to speculate that frondoside A might also activate a similar gut/brain axis.

## 15. Conclusions

Frondoside A has potent anti-cancer effects in all solid malignancy, lymphoma, and leukemia cell types investigated to date. Frondoside A causes growth inhibition, induction of apoptosis inhibition of migration, invasion and metastases, and blocks angiogenesis. The effects of frondoside A are mediated by inhibition of PAK1 and perhaps other mechanisms. Frondoside A potentiates the effects of conventional therapeutic agents, such as paclitaxel, cisplatin, and gemcitabine in several different cancer types. Over a fairly broad therapeutic range in experimental animals, frondoside A is well tolerated and appears to have no toxicity on bone marrow, liver, kidney, or other tissues, and does not affect body weight. Frondoside A can be readily produced from the waste-stream of certain sea cucumber processing; however, it could also be produced from cell culture of the skin from the source organism or conceivably by chemical synthesis [61]. Frondoside A may be valuable in the treatment of a wide range of malignancies either as a single agent or in combination with other therapeutics.

## Figures and Tables

**Table 1:** Effect of frondoside A on viability of different cancer cell lines reported in the literature.

Cell Line	Cancer Origin	Approximate IC_50_ µM	Hours Treated	Notes	Ref.
MiaPaca-2	Pancreas	0.5	24		[23]
AsPC-1	Pancreas	1.0	24		[25]
S2013	Pancreas	1.0	24		[25]
MDA-MB-231	Breast	1.2	48	Triple receptor negative	[13]
MCF-10A	Breast	5.0	48	Non-Malignant	[13]
66.1	Breast	0.5	24		[14]
MDA-MB-231	Breast	0.3	24	Three-dimensional culture	[17]
MDA-MB-435	Breast	2.5	24		[16]
MCF-7	Breast	2.0	24		[16]
LNM35	Lung	1.5	24	Met Sub-line of NCI-H460	[16]
A549	Lung	2.5	24		[16]
NCI-H460	Lung	2.5	24	Luciferase expressing cells	[16]
LNM35	Lung	0.6	72		[34]
HepG2	Liver	1.5	24		[16]
DLD-1	Colon	1.2	48		[23]
PC-1	Prostate	0.3	48		[23]
PC-3	Prostate	1.3	48		[18]
DU145	Prostate	1.0	48		[18]
LNCaP	Prostate	0.3	48		[18]
22Rv1	Prostate	0.1	48		[18]
VCaP	Prostate	0.2	48		[18]
MRC-9	Fibroblast	4.5	48	Non-Malignant	[18]
HEK293	Embryonic Kidney	1.9	48	Non-Malignant	[18]
HUVEC	Umbilical Vascular Endothelial	1.6	48	Non-Malignant	[18]
HT-1197	Bladder	2.3	48		[19]
486p	Bladder	1.1	48		[19]
RT4	Bladder	0.6	48		[19]
RT112	Bladder	0.5	48		[19]
T24	Bladder	1.5	48		[19]
TCC-SUP	Bladder	1.1	48		[19]
BL-2	Burkitt Lymphoma	0.2	48		[20]
CA46	Burkitt Lymphoma	0.2	48		[20]
Daudi	Burkitt Lymphoma	0.2	48		[20]
Raji	Burkitt Lymphoma	0.5	48		[20]
DG-75	Burkitt Lymphoma	0.2	48		[20]
EB1	Burkitt Lymphoma	0.6	48		[20]
Namalwa	Burkitt Lymphoma	0.2	48		[20]
Ramos	Burkitt Lymphoma	0.1	48		[20]
HL-60	Promyelocytic Leukemia	0.5	24		[12]
CCRF-CEM	T-Lymphoblastic Leukemia	1.5	48		[21]
THP-1	Monocytic Leukemia	3.0	48		[21]
HL-60	Promyelocytic Leukemia	2.5	48		[21]
NNCIT	Metastatic Germ Cell Tumor	0.5	Not reported	Cisplatin-resistant sublines equally sensitive	[24]
2102EP	Metastatic Germ Cell Tumor	0.5			[24]

**Table 2:** Effects of frondoside A *in vivo* in different mouse cancer models.

Cell Line	Cancer Origin	Dose	Outcome	Ref.
AsPC-1	Pancreas	10 µg/kg/day	Tumor size 56% of control at 32 days	[11]
AsPC-1	Pancreas	100 µg/kg/day + gemcitabine	Tumor size 13% of control at 30 days and combination had greater effect than either drug alone	[23]
S2013	Pancreas	100 µg/kg/day + gemcitabine	Tumor size 21% of control at 30 days and combination had greater effect than either drug alone	[23]
MDA-MB-231	Breast	100 µg/kg/day	Tumor size 4% of control at 27 days	[13]
LNM35	Lung	10 µg/kg/day	Tumor size 56% of control at 25 days	[16]
LNM35	Lung	1000 µg/kg/day	Tumor size 55% of control at 25 days	[16]
LNM35	Lung	100 µg/kg/day + cisplatin	Tumor size 32% of control at 10 days and combination had greater effect than either drug alone	[16]
PC-3	Prostate	100 µg/kg/day	Tumor size 58% of control at 30 days and reduced number of lung metastases	[18]
DU145	Prostate	800 µg/kg/day	Tumor size 47% of control at 25 days, abolished lung metastases and reduced circulating tumor cells	[18]

## References

[1] Adrian T.E. (2007). Novel marine-derived anti-cancer agents. Curr. Pharm. Des..

[2] Correia-da-Silva M., Sousa E., Pinto M.M.M., Kijjoa A. (2017). Anticancer and cancer preventive compounds from edible marine organisms. Semin. Cancer Biol..

[3] Tang W. (1987). Chinese medicinal materials from the sea. Abstr. Chin. Med..

[4] Pangestuti R., Arifin Z. (2017). Medicinal and health benefit effects of functional sea cucumbers. J. Trad. Complim. Med..

[5] Guo Y., Ding Y., Xu F., Liu B., Kou Z., Xiao W., Zhu J. (2015). Systems pharmacology-based drug discovery for marine resources: An example using sea cucumber (Holothurians). J. Ethnopharmacol..

[6] Li Y.X., Himaya S.W., Kim S.K. (2013). Triterpenoids of marine origin as anti-cancer agents. Molecules.

[7] Wargasetia T.L., Widodo (2017). Mechanisms of cancer cell killing by sea cucumber-derived compounds. Investig. New Drugs.

[8] Janakiram N.B., Mohammed A., Rao C.V. (2015). Sea Cucumbers Metabolites as Potent Anti-Cancer Agents. Mar. Drugs.

[9] Park J.I., Bae H.R., Kim C.G., Stonik V.A., Kwak J.Y. (2014). Relationships between chemical structures and functions of triterpene glycosides isolated from sea cucumbers. Front. Chem..

[10] Yu S., Ye X., Huang H., Peng R., Su Z., Lian X.Y., Zhang Z. (2015). Bioactive sulfated saponins from sea cucumber *Holothuria moebii*. Planta Med..

[11] Li X., Roginsky A.B., Ding X.Z., Woodward C., Collin P., Newman R.A., Bell R.H., Adrian T.E. (2008). Review of the apoptosis pathways in pancreatic cancer and the anti-apoptotic effects of the novel sea cucumber compound, Frondoside A. Ann. N. Y. Acad. Sci..

[12] Jin J.O., Shastina V.V., Shin S.W., Xu Q., Park J.I., Rasskazov V.A., Avilov S.A., Fedorov S.N., Stonik V.A., Kwak J.Y. (2009). Differential effects of triterpene glycosides, frondoside A and cucumarioside A2-2 isolated from sea cucumbers on caspase activation and apoptosis of human leukemia cells. FEBS Lett..

[13] Al Marzouqi N., Iratni R., Nemmar A., Arafat K., Al Sultan M.A., Yasin J., Collin P., Mester J., Adrian T.E., Attoub S. (2011). Frondoside A inhibits human breast cancer cell survival, migration, invasion and the growth of breast tumor xenografts. Eur. J. Pharmacol..

[14] Ma X., Kundu N., Collin P.D., Goloubeva O., Fulton A.M. (2012). Frondoside A inhibits breast cancer metastasis and antagonizes prostaglandin E receptors EP4 and EP2. Breast Cancer Res. Treat..

[15] Park S.Y., Kim Y.H., Kim Y., Lee S.J. (2012). Frondoside A has an anti-invasive effect by inhibiting TPA-induced MMP-9 activation via NF-κB and AP-1 signaling in human breast cancer cells. Int. J. Oncol..

[16] Attoub S., Arafat K., Gélaude A., Al Sultan M.A., Bracke M., Collin P., Takahashi T., Adrian T.E., De Wever O. (2013). Frondoside A suppressive effects on lung cancer survival, tumor growth, angiogenesis, invasion, and metastasis. PLoS ONE.

[17] Kundu N., Ma X., Kochel T., Goloubeva O., Staats P., Thompson K., Martin S., Reader J., Take Y., Collin P., Fulton A. (2014). Prostaglandin E receptor EP4 is a therapeutic target in breast cancer cells with stem-like properties. Breast Cancer Res. Treat..

[18] Dyshlovoy S.A., Menchinskaya E.S., Venz S., Rast S., Amann K., Hauschild J., Otte K., Kalinin V.I., Silchenko A.S., Avilov S.A. (2016). The marine triterpene glycoside frondoside A exhibits activity *in vitro* and *in vivo* in prostate cancer. Int. J. Cancer.

[19] Dyshlovoy S.A., Madanchi R., Hauschild J., Otte K., Alsdorf W.H., Schumacher U., Kalinin V.I., Silchenko A.S., Avilov S.A., Honecker F. (2017). The marine triterpene glycoside frondoside A induces p53-independent apoptosis and inhibits autophagy in urothelial carcinoma cells. BMC Cancer.

[20] Dyshlovoy S.A., Rast S., Hauschild J., Otte K., Alsdorf W.H., Madanchi R., Kalinin V.I., Silchenko A.S., Avilov S.A., Dierlamm J. (2017). Frondoside A induces AIF-associated caspase-independent apoptosis in Burkitt lymphoma cells. Leuk. Lymphoma.

[21] Sajwani F.H., Collin P., Adrian T.E. (2017). Frondoside A potentiates the effects of conventional therapeutic agents in acute leukemia. Leukem. Res..

[22] Aminin D.L., Menchinskaya E.S., Pisliagin E.A., Silchenko A.S., Avilov S.A., Kalinin V.I. (2015). Anticancer activity of sea cucumber triterpene glycosides. Mar. Drugs.

[23] Adrian T.E., Collin P. (2005). Anticancer Glycoside Compounds. U.S. Patent.

[24] Alsdorf W.H., Dyshlovoy S., Otte K., Hausschild J., Bokemeyer C., Honecker F., von Amsberg G. (2016). Cytotoxic activity and molecular mechanisms of action of the marine triterpene glycoside frondoside A in germ cell tumors. Oncol. Res. Treat..

[25] Al Shemaili J., Mensah-Brown E., Parekh K., Thomas S.A., Attoub S., Hellman B., Nyberg F., Adem A., Collin P., Adrian T.E. (2014). Frondoside A enhances the antiproliferative effects of gemcitabine in pancreatic cancer. Eur. J. Cancer.

[26] Al Shemaili J., Parekh K.A., Newman R.A., Hellman B., Woodward C., Adem A., Collin P., Adrian T.E. (2016). Pharmacokinetics in Mouse and Comparative Effects of Frondosides in Pancreatic Cancer. Mar. Drugs.

[27] Silchenko A.S., Avilov S.A., Kalinin V.I., Kalinovsky A.I., Dmitrenok P.S., Fedorov S.N., Stepanov V.G., Dong Z., Stonik V.A. (2008). Constituents of the sea cucumber Cucumaria okhotensis. Structures of okhotosides B1-B3 and cytotoxic activities of some glycosides from this species. J. Nat. Prod..

[28] Aminin D.L., Agafonova I.G., Kalinin V.I., Silchenko A.S., Avilov S.A., Stonik V.A., Collin P.D., Woodward C. (2008). Immunomodulatory properties of frondoside A, a major triterpene glycoside from the North Atlantic commercially harvested sea cucumber Cucumaria frondosa. J. Med. Food.

[29] Aminin D.L., Koy C., Dmitrenok P.S., Müller-Hilke B., Koczan D., Arbogast B., Silchenko A.A., Kalinin V.I., Avilov S.A., Stonik V.A. (2009). Immunomodulatory effects of holothurian triterpene glycosides on mammalian splenocytes determined by mass spectrometric proteome analysis. J. Proteomics.

[30] Aminin D.L., Silchenko A.S., Avilov S.A., Stepanov V.G., Kalinin V.I. (2009). Cytotoxic action of triterpene glycosides from sea cucumbers from the genus Cucumaria on mouse spleen lymphocytes. Inhibition of nonspecific esterase. Nat. Prod. Commun..

[31] Aminin D.L., Silchenko A.S., Avilov S.A., Stepanov V.G., Kalinin V.I. (2010). Immunomodulatory action of monosulfated triterpene glycosides from the sea cucumber *Cucumaria okhotensis*: Stimulation of activity of mouse peritoneal macrophages. Nat. Prod. Commun..

[32] Holt D.M., Ma X., Kundu N., Collin P.D., Fulton A.M. (2012). Modulation of host natural killer cell functions in breast cancer via prostaglandin E2 receptors EP2 and EP4. J. Immunother..

[33] Kovalchuk S.N., Kozhemyako V.B., Atopkina L.N., Silchenko A.S., Avilov S.A., Kalinin V.I., Rasskazov V.A., Aminin D.L. (2006). activity of triterpene glycosides in yeast two-hybrid assay. J. Steroid Biochem. Mol. Biol..

[34] Mazeĭka A.N., Popov A.M., Kalinin V.I., Avilov S.A., Sil’chenko A.S., Kostetskiĭ E. (2008). Complexation between triterpene glycosides of holothurians and cholesterol is the basis of lipid-saponin carriers of subunit protein antigens. Biofizika.

[35] Menchinskaya E.S., Aminin D.L., Avilov S.A., Silchenko A.S., Andryjashchenko P.V., Kalinin V.I., Stonik V.A. (2013). Inhibition of tumor cells multidrug resistance by cucumarioside A2-2, frondoside A and their complexes with cholesterol. Nat. Prod. Commun..

[36] Nguyen B.C.Q., Yoshimura K., Kumazawa S., Tawata S., Maruta H. (2017). Frondoside A from sea cucumber and nymphaeols from *Okinawa propolis*: Natural anti-cancer agents that selectively inhibit PAK1 *in vitro*. Drug Discov. Ther..

[37] Leist M., Jaattela M. (2001). Four deaths and a funeral: From caspases to alternative mechanisms. Nat. Rev. Mol. Cell Biol..

[38] Sperandio S., de Belle I., Bredesen D.E. (2000). An alternative, nonapoptotic form of programmed cell death. Proc. Natl. Acad. Sci. USA.

[39] Turmaine M., Raza A., Mahal A., Mangiarini L., Bates G.P., Davies S.W. (2000). Nonapoptotic neurodegeneration in a transgenic mouse model of Huntington’s disease. Proc. Natl. Acad. Sci. USA.

[40] Lee C.Y., Baehrecke E.H. (2001). Steroid regulation of autophagic programmed cell death during development. Development.

[41] Holler N., Zaru R., Micheau O., Thome M., Attinger A., Valitutti S., Bodmer J.L., Schneider P., Seed B., Tschopp J. (2000). Fas triggers an alternative, caspase-8-independent cell death pathway using the kinase RIP as effector molecule. Nat. Immunol..

[42] Los M., Mozoluk M., Ferrari D., Stepczynska A., Stroh C., Renz A., Herceg Z., Wang Z.Q., Schulze-Osthoff K. (2002). Activation and caspase-mediated inhibition of PARP: A molecular switch between fibroblast necrosis and apoptosis in death receptor signaling. Mol. Biol. Cell.

[43] Vercammen D., Brouckaert G., Denecker G., Van de Craen M., Declercq W., Fiers W., Vandenabeele P. (1998). Dual signaling of the Fas receptor: Initiation of both apoptotic and necrotic cell death pathways. J. Exp. Med..

[44] Denecker G., Vercammen D., Steemans M., Vanden Berghe T., Brouckaert G., Van Loo G., Zhivotovsky B., Fiers W., Grooten J., Declercq W., Vandenabeele P. (2001). Death receptor-induced apoptotic and necrotic cell death: Differential role of caspases and mitochondria. Cell Death Differ..

[45] Foghsgaard L., Wissing D., Mauch D., Lademann U., Bastholm L., Boes M., Elling F., Leist M., Jäättelä M. (2001). Cathepsin B acts as a dominant execution protease in tumor cell apoptosis induced by tumor necrosis factor. J. Cell Biol..

[46] Schulze-Osthoff K., Bakker A.C., Vanhaesebroeck B., Schulze-Osthoff K., Bakker A.C., Vanhaesebroeck B., Beyaert R., Jacob W.A., Fiers W. (1992). Cytotoxic activity of tumor necrosis factor is mediated by early damage of mitochondrial functions. Evidence for the involvement of mitochondrial radical generation. J. Biol. Chem..

[47] McCarthy N.J., Whyte M.K., Gilbert C.S., Evan G.I. (1997). Inhibition of Ced3/ICE-related proteases does not prevent cell death induced by oncogenes, DNA damage, or the Bcl-2 homologue Bak. J. Cell Biol..

[48] Borner C., Monney L. (1999). Apoptosis without caspases: An inefficient molecular guillotine?. Cell Death Differ..

[49] Déas O., Dumont C., MacFarlane M., Rouleau M., Hebib C., Harper F., Hirsch F., Charpentier B., Cohen G.M., Senik A. (1998). Caspase-independent cell death induced by antiCD2 or staurosporine in activated human peripheral T lymphocytes. J. Immunol..

[50] Miller T.M., Moulder K.L., Knudson C.M., Creedon D.J., Deshmukh M., Korsmeyer S.J., Johnson E.M. (1997). Bax deletion further orders the cell death pathway in cerebellar granule cells and suggests a caspase-independent pathway to cell death. J. Cell Biol..

[51] Xiang J., Chao D.T., Korsmeyer S.J. (1996). BAX-induced cell death may not require interleukin 1 beta-converting enzyme-like proteases. Proc. Natl. Acad. Sci. USA.

[52] Daugas E., Susin S.A., Zamzami N., Ferri K.F., Irinopoulou T., Larochette N., Prévost M.C., Leber B., Andrews D., Penninger J., Kroeme G. (2000). Mitochondrionuclear translocation of AIF in apoptosis and necrosis. FASEB J..

[53] Arnoult D., Tatischeff I., Estaquier J., Girard M., Sureau F., Tissier J.P., Grodet A., Dellinger M., Traincard F., Kahn A. (2001). On the evolutionary conservation of the cell death pathway: Mitochondrial release of an apoptosis-inducing factor during *Dictyostelium discoideum* cell death. Mol. Biol. Cell.

[54] Al Shemaili J., Parekh K., Thomas S.A., Kelly D.L., Ding X.Z., Attoub S., Collin S.P., Adrian T.E. (2013). Studies on the Mechanism of Action of Frondoside A in Pancreatic Cancer. Pancreatology.

[55] Kumar R., Gururaj A.E., Barnes C.J. (2006). p21-Activated kinases in cancer. Nat. Rev. Cancer.

[56] Rane C., Minden A. (2014). P21 activated kinases: Structure, regulation, and functions. Small GTPases.

[57] Wang Z., Jia G., Li Y., Liu J., Luo J., Zhang J., Xu G., Chen G. (2017). Clinicopathological signature of p21-activated kinase 1 in prostate cancer and its regulation of proliferation and autophagy via the mTOR signaling pathway. Oncotarget.

[58] Nheu T., He H., Hirokawa Y., Walker F., Wood J., Maruta H. (2004). PAK is essential for RAS-induced upregulation of cyclin D1 during the G1 to S phase transition. Cell Cycle.

[59] Huynh N., Wang K., Yim M., Dumesny C.J., Sandrin M.S., Baldwin G.S., Nikfarjam M., He H. (2017). Depletion of p21-ctivated kinase 1 up-regulates the immune system of APC∆14/+ mice and inhibits intestinal tumorigenesis. BMC Cancer.

[60] Dou Y., Luo J., Wu X., Wei Z., Tong B., Yu J., Wang T., Zhang X., Yang Y., Yuan X. (2018). Curcumin attenuates collagen-induced inflammatory response through the “gut-brain axis”. J. Neuroinflamm..

[61] Gomes N.G., Dasari R., Chandra S., Kiss R., Kornienko A. (2016). Marine Invertebrate Metabolites with Anticancer Activities: Solutions to the “Supply Problem”. Mar. Drugs.

